# Epidemiological-clinical profile and mortality in patients coinfected with *Trypanosoma cruzi*/HIV: experience from a Brazilian reference center

**DOI:** 10.1590/0037-8682-0240-2022

**Published:** 2022-10-21

**Authors:** Alejandro Marcel Hasslocher-Moreno, Andréa Silvestre de Sousa, Sergio Salles Xavier, Fernanda de Souza Nogueira Sardinha Mendes, Estevão Portela Nunes, Beatriz Gilda Jegerhorn Grinsztejn, Mauro Felippe Felix Mediano

**Affiliations:** 1 Fundação Oswaldo Cruz, Instituto Nacional de Infectologia Evandro Chagas, Rio de Janeiro, RJ, Brasil.; 2 Universidade Federal do Rio de Janeiro, Faculdade de Medicina, Rio de Janeiro, RJ, Brasil.

**Keywords:** Chagas disease, Trypanosoma cruzi, HIV, AIDS, Coinfection, Mortality

## Abstract

**Background:**

The recent urbanization of Chagas disease (CD) has contributed to a greater risk of coexistence with human immunodeficiency virus (HIV) and AIDS.

**Methods:**

This retrospective observational study included patients who were followed at INI-Fiocruz between July 1986 and October 2021. All patients underwent an assessment protocol that included sociodemographic profile, epidemiological history, and clinical evaluation. Descriptive data analyses included reports of the medians and frequencies of variables of interest. Differences in medians between groups were tested using the Mann-Whitney *U* test. Differences in frequency were tested using Fisher's exact test.

**Results:**

Among 2201 patients, 11 (0.5%) were identified with *Trypanosoma cruzi*/HIV coinfection. Of these, 63.6% were women with a median age of 51.0 years old. Two patients had the indeterminate form of CD, six had the cardiac form, two had the digestive form and one had the cardio-digestive form. Half of the patients were undergoing antiretroviral treatment at the time of coinfection diagnosis with a median CD4+ count of 350 cells/μL and a viral load of 1500 copies/μL. Four patients underwent a xenodiagnosis test at coinfection diagnosis, which all yielded positive results; two of them presented high parasitemia under the risk of reactivation. Prophylaxis for CD reactivation was administered to four patients; two with ketoconazole and two with benznidazole. Six patients died after a median follow-up of 22.5 months, with AIDS being the most common cause of death. Only one case of reactivation was observed.

**Conclusions:**

Early diagnosis and prompt treatment of CD reactivation dramatically reduced mortality. Identification of *Trypanosoma cruzi*/HIV co-infection is crucial to planning a close follow-up of coinfected patients.

## INTRODUCTION

Chagas disease (CD) is a neglected tropical disease caused by *Trypanosoma cruzi* infection. CD remains a serious public health problem in Latin America. Data from the World Health Organization estimate that 6-7 million people worldwide are infected with *T. cruzi* in 21 Latin American countries. In addition, numerous immigrants with CD have moved in recent years to non-endemic countries of the Northern Hemisphere, constituting a new challenge^1^. Brazilian socioeconomic changes in recent decades have promoted the urbanization of CD, a factor that also modifies its epidemiological profile^2^. Concomitantly, human immunodeficiency virus (HIV) has emerged in recent decades, predominantly in large urban centers where patients with CD are currently concentrated^3^. Therefore, the current geographic and social context may favor *T. cruzi*/HIV coinfection^4^.

In 2019, 41,909 new cases of HIV and 37,308 cases of acquired immunodeficiency syndrome (AIDS) were diagnosed in Brazil, with a total of 1,011,617 cases from 1980 to June 2020. There has been a recent decrease in the AIDS detection rate in Brazil, from 21.9/100,000 inhabitants in 2012 to 17.8/100,000 inhabitants in 2019, representing a decrease of 18.7%. This reduction was more pronounced since the “treatment for all” recommendations were implemented in December 2013^5^.

Data from the 2nd Brazilian Consensus on Chagas Disease estimate that in Brazil there were between 1,365,000 and 3,213,000 people with CD in 2020^6^. The Brazilian Ministry of Health^7^ estimates 16,100 cases of coinfection with a prevalence of 1.3-5% for *T. cruzi* infection in a population living with HIV^8^ ^,^ ^9^. Currently, considering the potential epidemiological risk contexts, the *T. cruzi* test is recommended among HIV-infected subjects^7^. For epidemiological surveillance purposes it is also noteworthy that since 2004, CD reactivation has been included in the list of AIDS defining illnesses and is considered an important marker of definitive Chagasic meningoencephalitis and Chagasic myocarditis diagnosis^10^. In individuals previously infected with *T. cruzi*, HIV infection usually occurs via sexual transmission as the main route. The expected trend in Brazil is a progressive decrease in cases of *T. cruzi*/HIV coinfection, since patients with CD are aging^11^. 

The first guideline for the clinical management of *T. cruzi*/HIV coinfection was published in 2006^12^, and additional guidelines and manuals were published^13^ ^,^ ^14^. In 2011, a systematic review included 83 articles with 291 cases of *T. cruzi*/HIV coinfection, with most studies describing clinical cases or case series^15^. Few studies have examined the clinical-epidemiological profiles of these patients^8^ ^,^ ^9^ ^,^ ^16^ ^-^ ^18^. Similar to other infectious diseases, *T. cruzi* can act an opportunistic microorganism in immunosuppressed individuals. Although the first case of *T. cruzi*/HIV coinfection was reported in the 1980s^19^, its frequency and eventual CD reactivation rates, as well as the clinical and laboratory profile of coinfected patients, their survival times and mortality rates are still underreported^20^ ^,^ ^21^. Our study aimed to describe the prevalence of *T. cruzi*/HIV coinfection in a cohort of patients regularly followed up at a Brazilian referral center for infectious diseases, as well as their epidemiological and clinical characteristics, the incidence of CD reactivation, mortality rate, and main causes of death. 

## METHODS

This retrospective observational study included patients followed up at the outpatient clinic of the Evandro Chagas National Institute of Infectious Diseases (INI) from the Oswaldo Cruz Foundation (Fiocruz), in Rio de Janeiro, Brazil. The diagnosis of CD was confirmed when two simultaneous serological techniques, indirect immunofluorescence (IFA) and enzyme linked immunosorbent assay (ELISA) yielded reactive results. Some patients were also subjected to parasitological evaluation through xenodiagnosis (xeno) as recommended by Cerisola et al.^22^. Qualitative and quantitative xeno-analyses were performed. Forty triatomine nymphs were examined after 45 days of feeding. Qualitative xeno was considered positive when at least one triatomine nymph tested was positive. The quantitative xeno analysis was based on the percentage of positive nymphs. HIV was diagnosed via two consecutive positive ELISA tests (HIV1/HIV2 antigens) and confirmed by a positive immunoblot test (HIV1/HIV2 antigens). 

Data were collected from the electronic medical records of outpatients from July 1986 to October 2021. All patients underwent an initial assessment including epidemiological history (mode of CD and HIV transmission and time away from the CD endemic area), sociodemographic data (age, education level and race), time since the diagnosis of co-infection (before and after 1997, when highly active antiretroviral therapy [HAART] was introduced in Brazil), clinical form of CD, presence of comorbidities, etiological treatment of CD, antiretroviral therapy, prophylaxis for CD reactivation, death and related cause and laboratory evaluation (CD4+ cell count and viral load).

The routes of CD transmissions considered were vectorial, transfusional, congenital, and oral. Education level was categorized based on the number of years of formal study as illiterate, < 9 years, or > 9 years. Race was self-reported and classified as white, black, mixed, or indigenous. Clinical forms of chronic CD were classified according to the 2nd Brazilian Consensus on Chagas Disease^6^. Comorbidities included systemic arterial hypertension, diabetes mellitus, dyslipidemia, non Chagasic cardiomyopathy, and hepatitis B diagnosed at the time of coinfection. CD treatment included the use of benznidazole for 60 consecutive days. Antiretroviral therapy included the regimen indicated by the Brazilian Ministry of Health according to the time of treatment initiation. Prophylaxis for CD reactivation comprised the use of benznidazole or ketoconazole (according to theoretical knowledge at that time) for patients who had a positive xeno or CD4+ cells count of <350 cells/μL. Prophylactic therapy was administered until the CD4+ cells count exceeded 350 cells/μL. High parasitemia under risk of reactivation was defined as the detection of >20% of positive triatomine nymphs on quantitative xeno and the absence of symptoms and signs compatible with acute CD. Clinical reactivation of CD was defined by the detection of *T. cruzi* in peripheral blood through a direct blood smear test and the presence of symptoms and signs compatible with acute CD. CD4+ cells counts and viral loads were determined according to the follow-up protocol for patients with HIV, as indicated by the Brazilian Ministry of Health^23^. Data regarding deaths and related causes were obtained via review of the electronic medical records, online consultation of the mortality database system of the Department of Justice (TJ/RJ) and Department of Health (SES/RJ) from the State of Rio de Janeiro, and telephone contact with patients’ relatives when available.

### Data analysis

The statistical analyses were performed using Stata version 13.0 (StataCorp LLC, College Station, TX, USA). Variables of interest are presented descriptively as medians (with interquartile ranges) or frequencies (with percentages). Differences in medians between groups according to death status (survivors vs deceased) were tested using the Mann-Whitney *U* test. Differences in frequency were tested using Fisher’s exact test. Dummy variables were created for each category of race, educational level, CD transmission route, time away from the endemic area, HIV transmission route, clinical form of CD, and cardiac and digestive classification, allowing a direct comparison within categories according to death status. The significance level adopted for all tests was *p* <0.05.

### Ethics approval

The study protocol was approved by the INI-Fiocruz Research Ethics Committee (number CAAE: 35744820.8.0000.5262) on October 2, 2020 and was carried out in accordance with the 1964 Declaration of Helsinki and its later amendments. Considering the retrospective nature of the study, the need for informed consent was waived.

## RESULTS

Among the cohort of 2201 CD patients followed at INI-Fiocruz, between July 1986 and October 2021, 11 cases of *T. cruzi*/HIV coinfection were identified (prevalence of 0.5%). The main characteristics of *T. cruzi*/HIV patients are described in Table 1. The median age was 51.0 years and most patients were women (63.6%). White and mixed races were the most predominant (45.5% each) and a vast majority (91.0%) of patients had <9 years of formal education or were illiterate. Most patients had moved away from CD-endemic areas for more than 20 years and were migrants predominantly from Minas Gerais (45.4%) and Bahia (36.3%). The CD transmission mode was vectorial in all patients and the most common mode of HIV transmission was unprotected heterosexual contact (72.8%). Half of the patients were undergoing antiretroviral treatment at the time of coinfection diagnosis, all previously diagnosed with HIV infection, with a median CD4+ count of 350 cells/μL and a viral load of 1500 copies/μL. Twenty-five percent of the patients presented CD4+ levels <200 cells/μL. Most of patients had been diagnosed with coinfection after 1997, when HAART therapy was introduced in Brazil. CD was diagnosed first in 54.5% of the patients and the most common clinical form of CD was cardiac (54.6%), with equal distribution for stages A, B1, and B2 (28.6% for each stage). Among those with a digestive presentation (n=3), most had both megaesophagus and megacolon (66.7%). The frequency of comorbidities was 54.5% for systemic arterial hypertension and 45.5% for dyslipidemia. Only four patients had a xenodiagnosis test at coinfection diagnosis, which all yielded positive results; two of them presented with high parasitemia with a risk of reactivation. Only one case of confirmed clinical CD reactivation was observed (9.0%). Prophylaxis for CD reactivation at coinfection diagnosis was administered in four (36.3%) patients; two with ketoconazole and two with benznidazole. Six patients (54.5%) died after a median follow-up of 22.5 months, with AIDS being the most common cause of death (n=3, 50%). The patient with CD reactivation died 15 months after the diagnosis of *T. cruzi*/HIV co-infection due to neurotoxoplasmosis associated with AIDS. No major differences in epidemiological, sociodemographic, clinical, and laboratorial characteristics were observed between the groups according to death status (survivors vs deceased) (Table 2). A detailed description of each of the 11 *T. cruzi*/HIV coinfected patients is presented in Table 3. 

**TABLE:** 1: Epidemiological, sociodemographic, clinical, and laboratory characteristics of patients with *T. cruzi*/HIV coinfection followed at INI-Fiocruz (n=11).

Variables	Median (IQR) or n (%)
	Total
**Age at coinfection diagnosis, years**	51 (56-46)
Men	4 (36.4)
**Race**	
White	5 (45.5)
Mixed	5 (45.5)
Black	1 (9.0)
**Educational level**	
Illiterate	4 (36.4)
<9 years	6 (54.6)
>9 years	1 (9.0)
**CD transmission mode**	
Vectorial	11 (100.0)
Transfusional	0 (0.0)
Congenital	0 (0.0)
Oral	0 (0.0)
**Time away from endemic area**	
None	1 (9.0)
1-20 years	2 (18.2)
>20 years	8 (72.8)
Non-endemic area	0 (0.0)
**HIV transmission mode**	
Unprotected heterosexual contact	8 (72.8)
Unprotected homosexual contact	2 (18.2)
Contaminated objects	1 (9.0)
Antiretroviral therapy at coinfection diagnosis (n=10)	5 (50.0)
CD4+ at HIV diagnosis (n=8)	350 (133-562)
Viral load at HIV diagnosis (n=7)	1500 (0-59103)
CD4+ <200 at coinfection diagnosis (n=8)	2 (25.0)
Coinfection diagnosis before 1997	2 (18.2)
**CD clinical form at coinfection diagnosis**	
Indeterminate	2 (18.2)
Cardiac	6 (54.6)
Digestive	2 (18.2)
Cardio-digestive	1 (9.0)
**Classification of cardiac form at coinfection diagnosis (n=7)**	
Stage A	2 (28.6)
Stage B1	2 (28.6)
Stage B2	2 (28.6)
Stage C	1 (14.2)
**Classification of digestive form at coinfection diagnosis (n=3)**	
Megaesophagus	1 (33.3)
Megacolon	0 (0.0)
Megaesophagus and megacolon	2 (66.7)
Positive qualitative xenodiagnosis at coinfection diagnosis (n=4)	4 (100.0)
Quantitative xenodiagnosis at coinfection diagnosis (n=4)	16.3 (5-55.0)
Diagnosis of CD first	6 (54.5)
**Comorbidities at coinfection diagnosis**	
Systemic arterial hypertension	6 (54.5)
Diabetes mellitus	0 (0.0)
Dyslipidemia	5 (45.5)
Hepatitis B	1 (9.0)
Non-Chagasic Cardiomyopathy	0 (0.0)
**Antiparasitic drugs**	
Ketoconazole prophylaxis (200 mg once a day / 6 months)	2 (18.2)
Benznidazole prophylaxis (100 mg twice a day / 6 months)	2 (18.2)
Benznidazole treatment (100 mg twice a day / 2 months)	2 (18.2)
High parasitemia under risk of reactivation (n=5)	2 (40.0)
Clinical reactivation of CD	1 (9.0)
Death	6 (54.5)
**Cause of death (n=6)**	
CD reactivation	0 (0.0)
Chronic CD	1 (16.7)
AIDS	3 (50.0)
Other	2 (33.3)

**TABLE 2:** Characteristics of *T. cruzi*-HIV coinfection according to death status (survivors vs deceased).

Variables	Median (IQR) or n (%)		***p-value* (deceased vs survivors)**
	Survivors (n=5)	Deceased (n=6)	
**Age at coinfection diagnosis, years**	64 (48-66)	50.5 (46-58)	0.21
Sex			
Men	3 (60.0)	1 (16.7)	0.24
Women	2 (40.0)	5 (83.3)	
**Race**			
White	1 (20.0)	4 (66.7)	0.24
Mixed	4 (80.0)	1 (16.7)	0.08
Black	0 (0.0)	1 (16.7)	1.00
**Educational level**			
Illiterate	2 (40.0)	2 (33.3)	1.00
<9 years	2 (40.0)	4 (66.7)	0.57
>9 years	1 (20.0)	0 (0.0)	0.46
**CD transmission mode**			
Vectorial	5 (100.0)	6 (100.0)	1.00
**Time away from an endemic area**			
None	0 (0.0)	1 (16.7)	1.00
1-20 years	2 (40.0)	0 (0.0)	0.18
>20 years	3 (60.0)	5 (83.3)	0.55
Non-endemic area	0 (0.0)	0 (0.0)	1.00
**HIV transmission mode**			
Unprotected heterosexual contact	3 (60.0)	5 (83.3)	0.54
Unprotected homosexual contact	1 (20.0)	1 (16.7)	1.00
Contaminated objects	1 (20.0)	0 (0.0)	0.46
**Antiretroviral therapy (n=10)**			
No	0 (0.0)	2 (33.3)	0.47
Yes	4 (100.0)	4 (66.7)	
CD4+ at HIV diagnosis	448.5 (133-831), n=4	350 (198-396.5), n=4	0.56
Viral load at HIV diagnosis	12500 (0-42051.5), n=4	1500 (399-300000), n=3	0.48
**CD4+ <200 at coinfection diagnosis (n=8)**			
No	3 (75.0)	3 (75.0)	1.00
Yes	1 (25.0)	1 (25.0)	
**Time of coinfection diagnosis**			
Before 1997	0 (0.0)	2 (33.3)	0.46
After 1997	5 (100.0)	4 (66.7)	
**CD clinical form at coinfection diagnosis**			
Indeterminate	1 (20.0)	1 (16.7)	1.00
Cardiac	2 (40.0)	4 (66.7)	0.57
Digestive	1 (20.0)	1 (16.7)	1.00
Cardio-digestive	1 (20.0)	0 (0.0)	0.45
**Classification of cardiac form at coinfection diagnosis (n=7)**			
Stage A	0 (0.0)	2 (50.0)	0.43
Stage B1	1 (33.3)	1 (25.0)	1.00
Stage B2	1 (33.3)	1 (25.0)	1.00
Stage C	1 (33.3)	0 (0.0)	0.43
**Classification of digestive form at coinfection diagnosis (n=3)**			
Megaesophagus	0 (0.0)	1 (100.0)	0.33
Megacolon	0 (0.0)	0 (0.0)	1.00
Megaesophagus and megacolon	2 (100.0)	0 (0.0)	0.33
**Qualitative xenodiagnosis at coinfection diagnosis (n=4)**			
Negative	0 (0.0)	0 (0.0)	---*
Positive	0 (0.0)	4 (100.0)	
Quantitative xenodiagnosis at coinfection diagnosis (n=4)	---	16.3 (5-55.0)	---
**Diagnosis of CD or HIV**			
CD first	2 (40.0)	4 (66.7)	0.57
HIV first	3 (60.0)	2 (33.3)	
**Comorbidities at coinfection diagnosis**			
Arterial hypertension	3 (60.0)	3 (50.0)	1.00
Diabetes mellitus	0 (0.0)	0 (0.0)	---*
Dyslipidemia	3 (60.0)	2 (33.3)	0.57
Hepatitis B	0 (0.0)	1 (16.7)	1.00
Non-Chagasic cardiomyopathy	0 (0.0)	0 (0.0)	---*
**Antiparasitic drugs**			
No	4 (80.0)	1 (16.7)	0.08
Yes	1 (20.0)	5 (83.3)	
**High parasitemia under risk of reactivation (n=5)**			
No	0 (0.0)	3 (60.0)	---*
Yes	0 (0.0)	2 (40.0)	
**Clinical reactivation of CD**			
No	5 (100.0)	5 (83.3)	1.00
Yes	0 (0.0)	1 (16.7)	

* *p*-value not calculated due to a lack of cases in the exposure or outcome categories.

**TABLE 3:** Detailed description of each *T. cruzi*/HIV coinfected patient (n=11).

Patient	1	2	3	4	5	6	7	8	9	10	11
Year of coinfection diagnosis	1990	1991	1998	2001	2004	2006	2008	2009	2015	2017	2019
Age (years)	43	51	47	50	48	73	58	66	64	66	44
Sex	F	F	M	F	M	F	F	F	M	F	M
Race	Wh	Mi	Wh	Wh	Mi	Wh	Bl	Wh	Mi	Mi	Mi
Origin (Brazilian state)	BA	BA	MG	MG	PB	MG	MG	MG	BA	BA	CE
Clinical form	IND	DIG	CAR	CAR	IND	CAR	CAR	CAR	CAR+DIG	DIG	CAR
CD4+ at coinfection diagnosis	NA	443	350	350	216	46	NA	50	NA	981	681
Viral load at coinfection	NA	NA	399	1,500	25,000	300,000	NA	59,103	NA	0	0
Xeno CD	POS	NA	NA	POS	NEG	NA	NA	NA	NA	NA	NA
Q-xeno CD	2.5%	NA	NA	5%	NA	NA	NA	NA	NA	NA	NA
Xeno coinfection	POS	NA	POS	POS	NA	POS	NA	NA	NA	NA	NA
Q-xeno coinfection	27.5%	NA	5%	5%	NA	82.5%	NA	NA	NA	NA	NA
HAART therapy	NO	NO	YES	YES	NA	NO	NO	NO	YES	YES	YES
Antiparasitic drugs (prophylaxis)	KET	KET	NO	NO	NO	BZN	NO	BZN	NO	NO	NO
Antiparasitic drugs (treatment)	BZN	NO	BZN	BZN	NO	NO	NO	NO	NO	NO	NO
Xeno under antiparasitic drugs	NEG	NEG	NEG	NEG	NA	NA	NA	NA	NA	NA	NA
HP under risk of reactivation	YES	NO	NO	NO	NO	YES	NO	NO	NO	NO	NO
Clinical Reactivation	YES	NO	NO	NO	NO	NO	NO	NO	NO	NO	NO
Last CD4+ at follow-up	NA	937	556	NA	NA	NA	NA	470	597	899	988
Last viral load at follow-up	NA	0	4,700	NA	NA	NA	NA	0	0	0	0
Follow-up	Death	Death	Death	Death	NA	Death	Death	Alive	Alive	Alive	Alive
Cause of death	AIDS	AMI	Cancer	AIDS	NA	CD	AIDS	NA	NA	NA	NA

**CD:** Chagas disease; **NA:** not available; **F:** female; **M:** male; **Wh:** white; **Mi:** mixed; **Bl:** black; **IND:** indeterminate; **CAR:** cardiac; **DIG:** digestive; **CD4+:** CD4+ T cells/μL; **Viral load:** number of copies/μL: **Xeno CD:** xenodiagnosis at Chagas disease diagnosis; **Xeno HIV:** xenodiagnosis at the coinfection diagnosis; **Q-xeno:** quantitative xenodiagnosis/percentage of nymphs positive for *T. cruzi*; **HP:** high parasitemia; **HAART:** use of highly active antiretroviral therapy at the diagnosis of *T. cruzi*/HIV coinfection; **Xeno under antiparasitic drugs:** xenodiagnosis using ketoconazole or benznidazole; **AMI:** acute myocardial infarction; **BA:** Bahia; CE: Ceará; **MG:** Minas Gerais; **PB:** Paraíba.

## DISCUSSION

INI-Fiocruz is a reference center for infectious diseases that provides integral and multidisciplinary clinical care to patients. CD and HIV infections are among the various infectious diseases that are treated at the institution. Our study aimed to identify the prevalence of HIV infection in the INI-CD cohort. According to data from the Brazilian Health Surveillance Department, 444 cases of *T. cruzi*/HIV coinfection were documented in Brazil between January 2007 and August 2019^24^. However, few studies have presented clinical and epidemiological descriptions of these coinfection cases. A cross-sectional study carried out to investigate *T. cruzi*/HIV coinfection in a group of patients with CD followed up at a specialized service showed a prevalence of 0.65%. This study was conducted in Spain between 2004 and 2014, with a predominantly Bolivian population^17^. Another study, also conducted in Spain with a CD cohort of Bolivian migrants, found a *T. cruzi*/HIV coinfection prevalence of 0.41%^25^. In our study, which used a similar approach to identify HIV cases in a cohort of patients with CD, a *T. cruzi*/HIV coinfection prevalence of 0.50% was identified, which is very close to those reported in the Spanish studies. On the other hand, cross-sectional studies conducted to define the prevalence of CD among HIV patients have shown a higher prevalence. A study including HIV patients followed at a Brazilian hospital between 1994 and 2001 showed a *T. cruzi*/HIV coinfection prevalence of 1.3%^8^. Another study conducted in southern Brazil carried out an active search for CD in patients with HIV, and found *T. cruzi*/HIV coinfection prevalence of 5%^9^. In Spain, a study that evaluated the presence of tropical diseases in HIV infected immigrants identified a 2.6% prevalence of CD^26^. Another study that evaluated HIV-infected patients at a health center in Buenos Aires found an overall coinfection prevalence of 4.2%^27^. This significant difference in the prevalence of *T. cruzi*/HIV coinfection in relation to our study can be explained by methodological differences both in terms of study design and screened population.

In the present study, most patients were long-term residents of the metropolitan region of Rio de Janeiro who had moved away from CD-endemic areas for more than 20 years. They were migrants from four Brazilian states, predominantly Minas Gerais (45.4%) and Bahia (36.3%). These two states represent almost 50% of the INI CD cohort^28^. Similar data were shown in a study in which 45% of the patients were born in Bahia and Minas Gerais^16^. In our study, the mean age of the patients at the time of diagnosis of HIV infection varied according to the year of diagnosis of coinfection. Patients diagnosed in the 1990s and the early 2000s were between 40 and 50 years old, whereas those diagnosed after the mid-2000s predominated in the group aged 60 years. This condition reflects the aging of the INI CD cohort^28^. According to data from the Brazilian Health Surveillance Department, the mean age of coinfected patients was 40.3 years^24^. Studies conducted in Brazil that evaluated the mean age of coinfected patients between 1989 and 2005 reported a mean age of 38-43 years^8^ ^,^ ^16^. 

According to epidemiological history, all these patients were infected with *T. cruzi* during childhood. The vector route was the most probable in all patients, whereas unprotected heterosexual contact was the most common risk factor for HIV. Data from the Brazilian Health Surveillance Department showed a high prevalence of coinfection in men (70%)^24^. The INI-CD cohort showed a slight predominance of women, characterizing a balanced cohort in terms of sex^28^, while the INI-HIV cohort showed a high prevalence among males (71.2%)^29^. In our data, women predominated in the 1990s, while from 2000 onwards there was a balance between both sexes. These findings are not in line with data from most studies, which showed a prevalence in men ranging from 50-78%^9^ ^,^ ^16^ ^-^ ^18^. In only one study was the predominance of women higher (66%)^8^. Women with CD who were treated at our institution had low educational levels, received a minimum wage, and lived in high-risk violent communities. A study that evaluated gender differences among persons with HIV, admitted to a university reference center in São Paulo, Brazil, observed a predominance of married women with less education, denoting the particular social vulnerability exhibited by the female population^30^. Although white CD patients predominated in the INI-CD cohort^28^, in the present study there was a balance between white (45.4%) and mixed (45.4%) patients, similar to data from the Brazilian Health Surveillance Department', which indicated 42.4% of whites and 37.7% of mixed patients^24^. 

Regarding the clinical form of CD, most patients had the cardiac form, and one had heart failure. In most studies that evaluated the clinical form of CD, the indeterminate form predominated (49-65%)^8^ ^,^ ^16^ ^,^ ^17^, although a recent study of cases of multicentric network coinfection *T. cruzi*/HIV showed a balance between the indeterminate and cardiac forms^20^. The higher frequency of the cardiac form in our study may be explained by the age of the patients and the fact that they were treated at a tertiary care hospital. Among the three patients with megaesophagus, one developed asymptomatic esophageal candidiasis during the immunosuppression period, reversing this clinical picture after initiating HAART. The only case of reactivation occurred in a patient with the indeterminate form who presented acute myocarditis^31^. All patients had comorbidities, with a predominance of systemic arterial hypertension and dyslipidemia, and approximately one-third of the patients had more than one comorbidity. In a recent study, a high prevalence of metabolic syndrome was found in the INI-CD cohort with a high prevalence of systemic arterial hypertension and dyslipidemia^32^. 

Recently, a study that analyzed the major clinical characteristics and mortality rates in coinfection from a multicenter data base, showed the prognostic role of CD4+ cells in reactivation and mortality^20^. In our study, the mean CD4+ count was 389 cells/μL, with two patients having a count below 100 cells/μL. Other studies have reported a mean CD4+ count at the time of coinfection ranging from 103-294 cells/μL^8^ ^,^ ^16^ ^-^ ^18^. A study that analyzed the data of 241 patients showed that a lower CD4+ cells count at the time of coinfection diagnosis was independently associated with reactivation^20^. In the present study, the only case of reactivation that we identified occurred in 1990, and no CD4 cells count quantification was performed at that time.

Quantitative xeno was performed in four asymptomatic patients to assess *T. cruzi* parasitemia at the time of coinfection diagnosis, all of which yielded positive results. Direct microscopic tests to identify *T. cruzi* in peripheral blood were not performed concomitantly with xeno. In two of them, high parasitemia was observed in 27.5% and 82.5% of infected triatomine nymphs respectively, signifying high parasitemia under the risk of reactivation. Studies using qualitative xeno in patients in the chronic phase of CD, in the absence of immunosuppressive conditions, show varied positivity rates, according to the method used, ranging from 9 to 87.5%, although most of them have positive test rates from 40-50%^33^ ^,^ ^34^. In coinfected patients, quantitative xeno is more important than qualitative xeno, since it allows the assessment of the real risk of reactivation, with more infected triatomine nymphs associated with greater parasitemia. A study using quantitative xeno in patients with chronic CD without coinfection indicated that, in case of positivity, only up to 10% of the triatomine nymphs used had detectable *T. cruzi* ^35^. Another study that used quantitative xeno compared three groups of patients with chronic CD, *T. cruzi*/HIV infection without CD reactivation, and CD reactivation. The results showed that the level of parasites was lower in the chronic CD group and higher in coinfected patients, and 50% of the CD reactivated group had a much higher parasite load with more than 20% of the nymphs testing positive^36^. 

The Clinical Protocol and Therapeutics Guidelines for Infection Management for HIV in adults, from the Brazilian Ministry of Health, recommends considering therapy with trypanocidal drug in coinfected patients, even without evidence of symptoms, based on high parasitemia and low CD4+ cells levels, as well as in patients without documented reactivation but with persistent elevated parasitemia (quantitative xeno or PCR)^7^. There is no information regarding prophylaxis for reactivation of CD, although, in a case report involving a *T. cruzi*/HIV coinfected patient, the use of ketoconazole in the prophylaxis of reactivation was followed by a negative xeno result after 70 days of medication use^31^. In our study, six patients were treated with antiparasitic drugs and five of them had CD4 cell count information, two of them below 200 cells/μL. Among the patients who received etiologic treatment, four patients underwent prophylactic therapy for reactivation. The first two cases received ketoconazole (200 mg once a day), a drug that has no trypanocidal effect but suppresses parasitemia in experimental acute infection in animals^37^. In both cases, the response was a suppressive effect, as proven through negative xenos during the use of this drug. One of the patients, after six months, stopped using the medication on his own and presented CD reactivation, and was treated with benznidazole for 60 days. The other patient used ketoconazole for six months, with sufficient time for antiretroviral medications to improve the CD4+ count below cells count, and the use of ketoconazole could be discontinued. Another two patients received benznidazole (100mg twice a day), one of whom had a CD4+ less than 100 cells/μL. Of these two patients, one died six months after coinfection diagnosis, while receiving prophylactic benznidazole. The other patient received benznidazole for six months until immunity recovered. Two patients, with a CD4 cells count equal to 350 cells/μL and with quantitative xeno without evidence of high parasitemia under the risk of reactivation, were treated with benznidazole for 60 days, presenting negative post-treatment xeno. None of the four patients who received benznidazole experience adverse reaction. The xenos of the four patients who underwent prophylactic treatment to prevent CD reactivation were repeated during the use of this medication and all tests yielded negative results. 

The mean follow-up duration in our study was 132 months. Observational studies with coinfected patients signaled mean follow-up times between 35.8 and 65.5 months^8^ ^,^ ^16^ ^,^ ^17^ ^,^ ^25^. In our study, six (60%) of the 10 patients who were followed up died. The median age of patients who died was 50.5 years and 83.3% were women. Among those who died, three (50%) had a cause of death directly related to AIDS (neurotoxoplasmosis, atypical mycobacteriosis and bacterial pneumonia), one (16.7%) related to CD (sudden death), and two from other causes (one due to ischemic heart disease and one due to esophageal cancer). Deaths related to immunosuppression occurred in 2 patients who did not receive antiretroviral therapy and in 1 patient due to low adherence to treatment. The only patient who showed CD reactivation was treated with benznidazole, with complete remission of acute CD myocarditis, and later died of neurotoxoplasmosis^31^. In a study that compared 80 T. cruzi/HIV coinfected patients, with and without CD reactivation, deaths occurred in 55.5% of patients with reactivation as a result of central nervous system involvement^18^. A study of the epidemiological and clinical aspects of deaths related to *T. cruzi*/HIV coinfection in Brazil from 1999 to 2007 showed that AIDS was the underlying cause in 77.0% and CD in 17.6% of cases, with patients aged between 50-59 years accounting for 29.7% of deaths, and a predominance of men (51.4%)^38^. Two other studies that followed coinfected patients and assessed mortality found death rates of 26.4%^16^ and 55%^8^, respectively. In the first study, eight patients died as a result of CD, five due to CD reactivation and three due to sudden death. In a recent multicenter study that evaluated 241 coinfected patients, a 34.4% mortality rate was observed. The two leading causes of death were CD reactivation (28.9%) and opportunistic AIDS diseases (27.7%). The mortality rate due to chronic CD was 9.6%^20^.

 Our study had some limitations. The diagnosis of HIV/AIDS in the CD cohort was based on clinical suspicion, with the possibility of underestimating the prevalence of coinfection. Likewise, there was no active search for coinfection in the HIV/AIDS cohort, even when there was a previous epidemiological risk for CD. In addition, the patients in our study did not undergo evaluation or follow-up using real-time PCR assay. This test is more sensitive than blood culture, xeno and both tests combined^36^, and has been suggested for monitoring and identifying the risk of reactivation even before clinical symptoms occur^39^.

In conclusion, the prognosis of *T. cruzi*/HIV coinfection and CD reactivation was poor before the advent of HAART. In the current context of HIV diagnosis and treatment, early identification of *T. cruzi*/HIV coinfected patients is of paramount importance to ensure monitoring of their immune status and, in the event of immunosuppression, to initiate trypanocidal therapy to prevent the reactivation of CD. Two strategies are fundamental: the active search for CD in patients with HIV/AIDS with an epidemiological risk for *T. cruzi* infection, living in endemic or non-endemic areas, and the provision of HIV counseling and serology at the chronic longitudinal follow-up of CD patients.
